# Using Information and Communication Technologies to Prevent Suicide Among Secondary School Students in Two Regions of Chile: A Randomized Controlled Trial

**DOI:** 10.3389/fpsyt.2018.00236

**Published:** 2018-06-05

**Authors:** Franco Mascayano, Sara Schilling, Eric Tapia, Felipe Santander, María S. Burrone, Lawrence H. Yang, Rubén Alvarado

**Affiliations:** ^1^Department of Epidemiology, Mailman School of Public Health, Columbia University, New York City, NY, United States; ^2^Institute of Health Sciences, Universidad de O'Higgins, Rancagua, Chile; ^3^Faculty of Medicine, School of Public Health, Universidad de Chile, Santiago, Chile; ^4^Department of Social and Behavioral Sciences, College of Global Public Health, New York University, New York City, NY, United States

**Keywords:** suicide prevention, information and communication technologies, adolescents, randomized controlled trial, Latin America

## Abstract

**Background:** There is an increasing concern for addressing suicide among adolescents in Latin America. Recent mental health policies encourage the development and implementation of preventive interventions for suicide. Such initiatives, however, have been scarcely developed, even in countries with solid mental health services such as Chile. The use of information and communications technology (ICT) might contribute to create accessible, engaging, and innovative platforms to promote well-being and support for adolescents with mental health needs and suicide risk.

**Objective:** To evaluate a program based on ICT to prevent suicide and enhance mental health among adolescents in Chile. Method: A cluster randomized controlled trial (RCT) will be conducted including 428 high-school students aged 18–14 years in two regions of Chile. Study procedures will take place as follows: (1) design of the intervention model and creation of prototype; (2) selection and randomization of the participating public schools; (3) implementation of the 3-month intervention and evaluation at baseline, post-intervention period, and a 2-month follow-up. Suicidal ideation at the 2-month follow up is the primary outcome in this study. Secondary outcomes include negative psychological outcomes (e.g., stigma, depression, anxiety) as well as a number of protective psychological and social factors. Indicators regarding the study implementation will be also gathered. Discussion: Here we describe a novel program based on technological devices and aimed to target youth suicide in Chile. This is the first clinical trial of such a program in Latin America, and to our knowledge, the first of its kind in any middle income country.

**Trial Registration**: gov Identifier: NCT03514004

## Introduction

The World Health Organization reports that over 800,000 people die by suicide each year, and life-threatening behaviors are 10–20 times more frequent than completed suicides (1). Suicide prevalence varies throughout the lifespan, and for individuals between 15 and 29 years of age, it is the second leading cause of death globally, resulting in a massive burden for individuals, families, and communities at large. While statistics differ between countries and regions, it is estimated that 75% of all suicides occur in low-and-middle income countries (LMICs) (1).

Several high-income countries (HICs) have developed and implemented suicide prevention strategies. The effectiveness of such approaches, while promising, has yet to be fully established, and further research is needed (2). In LMICs, the knowledge gap is even wider—only a few interventions have been tested so far (3), in spite of the increasing prevalence rates recently reported (4).

In Latin America in particular, suicide rates have been rising over the past two decades (5), principally among adolescents and older adults, which poses a challenging and urgent problem for health services. Chile, for its part, has one of the highest rates of adolescent suicide worldwide (6), and addressing suicide has been named one of the principal health goals of the decade (7). Although a National Plan on Adolescent Suicide is already in place, initiatives specifically tailored to this population have been scarcely implemented.

The Plan, however, does highlight the importance of developing programs to prevent suicide through the promotion of self-esteem and self-efficacy, the strengthening of familial and community ties, and the construction of support networks among adolescents (8).

In this paper, we describe a cluster randomized controlled trial (RCT) of an intervention to prevent adolescent suicide, through a web-based platform and mobile application that will cultivate a community directed toward a goal of mutual support, in order to foster mental health protective factors and strengthen collaborative relationships among high school students, in two regions of Chile.

### Past interventions based on information and communications technologies to reduce suicide

Some have noted that information and communications technology (ICT)-based interventions—which use technologies such as desktop and laptop computers, hardware and software, mobile applications, and internet-based programs—may offer several advantages to address suicide among adolescent populations by (1) promoting an accessible, engaging, and collaborative learning and exchange environment for adolescents; (2) fostering creativity, self-expression, and collective generation of content; and (3) strengthening self-identity and the development of social connections, through the constitution of social networks and peer support groups (9, 10).

ICT interventions specifically designed to prevent suicide or address associated factors, such as depression, bullying, anxiety disorders, stress, and well-being, in adolescents have been piloted in High Income Countries (HICs), showing promising results in coverage, access, and cost-benefit effectiveness (11). Most of these programs, however, have only been tested in non-controlled trials, thus making it difficult to recommend their implementation on a larger scale or as a regular provision of public services (12). Additionally, suicidality is usually conceptualized as exclusion criteria in studies on ICT-based interventions for mental health problems which contributes to the lack of studies on ICT interventions for suicide risk.

In this regard, Lai et al. (12) carried out a systematic review of all internet-based interventions that have sought to prevent suicide or associated factors with a focus on analyzing the effectiveness of and what are possible barriers to these programs. Of the 13 studies that met inclusion criteria, only three were RCTs. The review also notes that it was not possible to determine an overall effect size, due to a lack of homogeneity with respect to the population, study design, evaluation methods, and intervention characteristics.

Of particular relevance is a large RCT conducted in seven European countries which evaluated a program, entitled “Supreme Project,” that was designed to promote protective factors in secondary school students through a series of online resources such as fact sheets, chats, forums, and games (13). Participants in the intervention group reported using the web platform almost every day and had a positive impression of its content and format. Main findings of the trial showed that the intervention group had decreased suicide risk and improved self-esteem (14). Nevertheless, differences between the control and intervention groups were not significant at follow-up.

Additional qualitative findings from the Supreme Project (14) indicate that the following practices should be considered in future suicide prevention programs: (1) the incorporation of adolescents as “peers;” (2) a gender-specific approach to tailor, and adapt contents; (3) culturally-sensitive adaptations for each strategy included in the program; (4) ongoing evaluations; and (5) the assessment of outcomes such as acceptability, satisfaction, and adoption, to inform the feasibility of implementing the intervention. These practices have strongly influenced our intervention proposal as detailed below.

### Using ICT-based interventions to prevent suicide in chile and latin america

It is estimated that only 45% of adolescents with suicidal risk in Chile have access to in—person treatment with a mental health professional (15). This gap is partially due to the limited availability of specialized mental health services for adolescents, lack of information about existing services, as well as adolescents' resistance to seek professional help, due to fear of being stigmatized by their peers, community or even their parents (16–18). It has been suggested that web-based interventions and/or mobile applications could be particularly suitable for adolescents, to overcome the aforementioned barriers to treatment, since ICTs provide easy access to valuable resources and reduce possible stigma associated with help-seeking and smental distress (19).

ICT-based interventions have special potential to be implemented in Chile (and elsewhere in Latin America), since the vast majority of the population has internet access and use of electronic devices—laptops, smartphones, tablets, etc.—is common across all social strata (20). Among adolescents in Chile, Internet use is even more intensive (90% connect at least once a day) (20). Adolescents, moreover, are not afraid to interact with others using new technologies in comparison to adults and older adults. Thus, leveraging technological solutions provides a strategic opportunity to reach young populations with an intervention that is suitable to the current forms of communication and interaction used frequently in their everyday lives (21). A further advantage of using this approach is that, if found to be effective, it can be easily disseminated, allowing for rapid adoption, and scale up at regional-level (22).

### The setting

The prevalence of adolescent suicide in Chile is 8.5 per 100,000 individuals, figures that have practically doubled since the 90s (1). Further, the rate of suicidal ideation is extremely elevated (30–60%) according to recent reports (15, 23). Among countries included in the Organization for Economic Co-operation and Development (OECD), Chile consistently presents one of the highest rates of suicide among adolescents (6). Though these prevalence rates have fallen slightly in recent years (1), Chile has yet to develop or implement a culturally relevant study to reduce youth suicide rates. This shortfall is representative of a general lack of evidence-based practices for youth mental health conditions, which is unfortunately common in most Latin American countries.

Nonetheless, a series of recent reforms on mental health, both at health service—and school-level, provide an advantageous scenario to develop, and sustain an intervention on suicide prevention for the youth in Chile. First, the Ministry of Health has announced that suicide among adolescents is a top priority and declared that the reduction of youth suicide rates by 10% is one of the top goals of the country's National Health Strategy for the decade 2011–2020 (7). Second, as noted above, a National Plan on Suicide was recently launched with the purpose of promoting competencies in the general population in terms of self-care, informed lifestyle decisions, and healthy environments that enable suicide reduction (8). Third, the development of preventive strategies to reduce self-destructive behavior, detect risk factors, and foster protective factors during adolescence (enhancing family support, social networks, and community resources) was recently proposed as a new component of the Plan (8). It has been recommended that part of such strategies should be anchored in school settings (24). Finally, these initiatives have been accompanied by an increase in the coverage and technical capacity of national mental health services, which have grown and strengthened in the last 15 years (25).

As noted above, the evidence for suicide prevention strategies is promising but still inconclusive. In addition, the major bulk of this evidence often pertains to interventions launched in HICs that cannot be implemented at a large scale in Low and Middle Income Countries (LMICs), such as Latin America countries, without local adaptation. Some of these interventions are costly and complex and/or are not suited to the service systems and sociocultural contexts of the region. A regionally-established evidence base would likely be more applicable and persuasive to stakeholders. On the other hand, an approach using ICT-based interventions might permit a rapid and feasible scale up, given the high use of electronic devices throughout the region. Such interventions could be an effective resource to prevent adolescent suicide, especially considering the characteristics, and idiosyncrasies of the target population. This proposal, therefore, seeks to provide and evaluate a program to prevent suicide among adolescents by leveraging technological solutions.

## Methods/design

### Trial design

**Figure 1:**
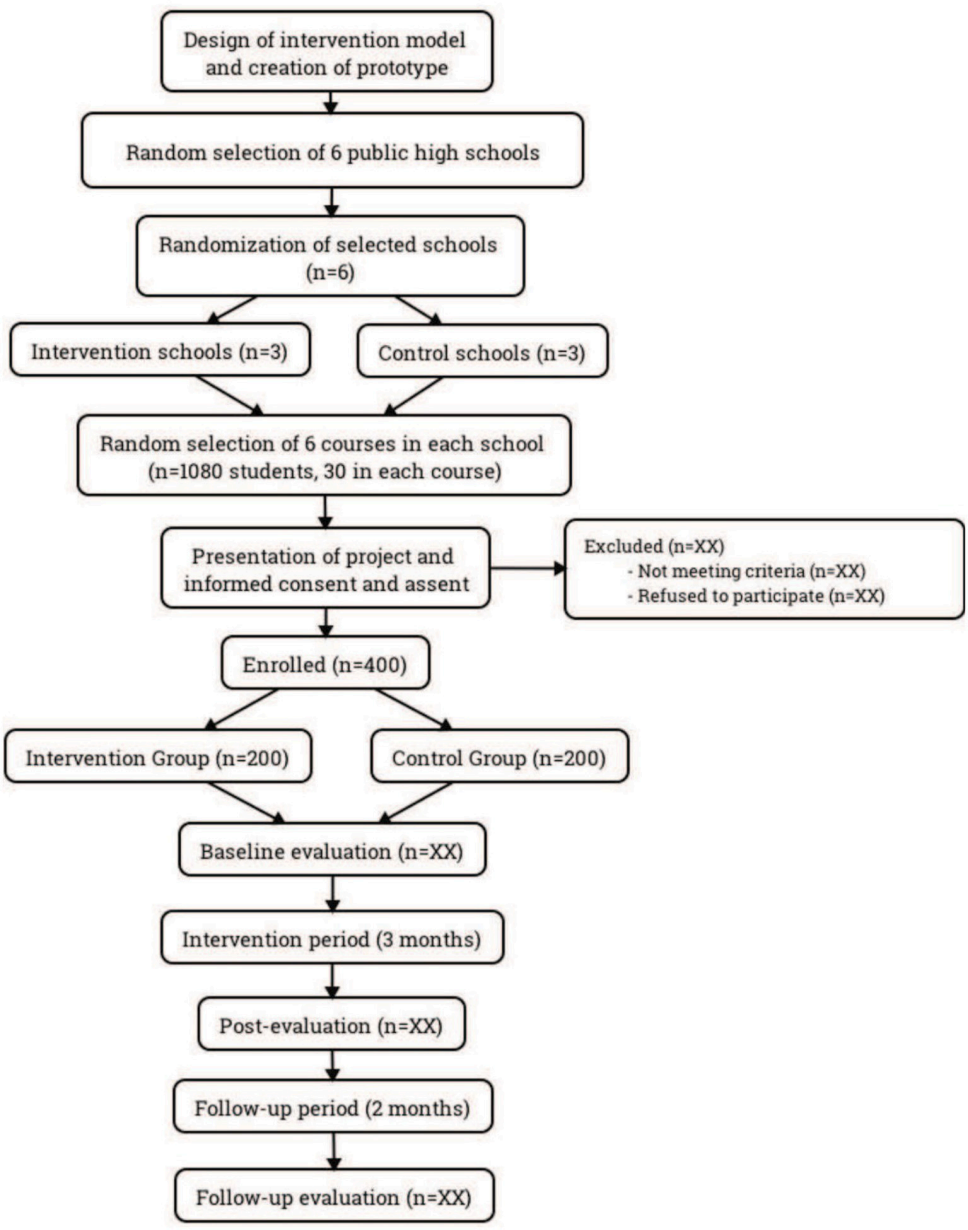
Flowchart of the cluster RCT.

This cluster RCT will be conducted in six public secondary schools in three municipalities, located in two regions of Chile: two municipalities from the Metropolitan Region (Region XIII, where the capital of Santiago is located) and one municipality in the Libertador General Bernardo O'Higgins Region (Region VI, to the south of Santiago, which is nearly 30% rural, in contrast to the Metropolitan Region, which is 3% rural) (26). The study procedure consists of six phases (see Figure 1): (1) design of the intervention model and creation of prototype; (2) selection of the 6 participating schools; (3) randomization of the secondary schools into 3 intervention sites and 3 control sites; (4) random selection of 6 courses in each school; (5) presentation of the study and informed consent for parents and assent for potential participants; and (6) implementation of the 3-month intervention and evaluation at baseline, post-intervention period, and a 2-month follow-up.

### Eligibility criteria for trial participants

The study population will consist of adolescents 14–18 years of age, who attend participating public high schools in the two regions of Chile mentioned above. These two regions were selected given the magnitude of their adolescent suicide prevalence rates (27) and feasibility considerations for the implementation of the study. Students who have visual or physical impairments that are incompatible with the intervention model will be excluded. Having a prior diagnosis of a mental disorder will not be considered among the exclusion criteria.

### Recruitment and screening

A random selection of schools and courses to participate in the study will take place in two stages. First, two public schools will be randomly selected from each of the three participating municipalities for a selection of a total of six schools. To do so, a list of all schools of each type will be drawn up for each municipality. A program will be used to randomly order the schools, and relevant authorities from the first school on each list will be contacted to invite them to participate in the study. This process will be supported by the respective authorities of the educational departments of the municipalities, who have already declared their interest and intent to collaborate with the study. Since participation is voluntary, schools will have the option to decline the invitation to participate, and if a school refuses participation, the next school on the list will be contacted until two schools from each municipality have agreed to participate. The first meeting with authorities will include a brief description of the study's objectives and procedures to clarify the substantive aspects of its implementation and the concrete support that will be required from them. Then, in each municipality, one of the schools will randomly be selected as the intervention site and the other will be the control site. Second, we will randomly select six courses from each of the six educational establishments. A recent study reported that there is an average of 30 students per course in public high schools in Chile (27), so it is estimated that these 36 courses will consist of approximately 1,080 students, who will be invited to participate in the study with their guardian's consent and their assent. Even with predicted losses due to lack of consent or assent, or meeting exclusion criteria, we expect to achieve at least 400 students to complete the recruitment and consent processes (see Figure 1), a large enough sample size to detect differences between groups as noted below.

### Randomization and treatment allocation

To present the study to potential participants, a teacher from the school, a member of the research team, and a peer-teenager (between 13 and 20 years of age), who is part of our team, will give a short presentation in both the intervention and control schools on the importance of mental health and personal and social well-being; in the intervention schools, they will also explain how the virtual community-based intervention can improve their well-being and serve to support other adolescents in the future. The role of the peer—teenager will be to speak from his or her experiences and feelings related to mental health conditions (depression, anxiety, suicidal ideation) that are familiar to adolescents (i.e., “this also happened to me”), and share part of the collaborative process behind the creation of the program. The participating teachers and teenager pairs will be trained by our research team and will proceed to invite new participants to the study.

The potential participants will then be asked to provide assent (with parent/guardian consent) to participate in the intervention or control group. All participants, regardless of whether they attend intervention or control schools, will be assessed on three occasions: at baseline, after the 3-month long intervention, and 2 months after the end of the intervention. The evaluations will be conducted through two approaches with some assessments being applied in person by trained mental health professionals and others that will be responded by the participants directly via the web platform that will be created for the present study. Participants will receive instructions on how to log into the system and will be notified at each of the three assessment points. Finally, all information collected will be stored digitally in the Data Coordinating Center located in the School of Public Health of Universidad de Chile in Santiago, Chile.

### Interventions

#### Description of design of intervention

In line with the best practices recommended by previous studies (14), peer-adolescents were integrated into our multi-disciplinary research team from the beginning of the study, to lead and advise the creation of the intervention model by playing a role as “experts by experience,” in order to ensure that the project is culturally relevant to the current context of high school students in Chile.

The “group of experts”—consisting of 7 adolescents, 13–20 years of age—were recruited and selected through a series of focus groups to reflect the diversity of adolescents in Chile, in terms of gender (4 women, 3 men), territorial origin (with members from northern, central, and southern regions), high school type (public, private, and subsidized schools), socioeconomic level, and sexual orientation. The peer-adolescents worked together with three members of the research team (ET a sociologist and public health researcher; SS a medical student and public health researcher; and FS a visual artist and graphic designer) over the course of 6 months to create the intervention model, on the basis of the literature and the adolescents' ideas, in light of their own experiences and those of their peers (28).

The ICT-based program utilizes a web-based platform and a mobile application to cultivate a virtual community to promote mental health protective factors, such as self-esteem and self-expression, and prevent adolescent suicide. To overcome the frequent barriers to help seeking, the program will provide rapid direct access to quality, evidence-based information and real-time assistance from a mental health professional; encourage habits that improve emotional and physical health; facilitate self-monitoring of mental health and personal progress; and promote social integration and participation in community-based activities.

The program is based on the principles of peer support and inclusivity, and as such, its name is “Project Clan,” in reference to a diverse group of individuals who come together for a common purpose in a welcoming environment. The privacy and anonymity of each “Clan member” will be respected, so that they feel free to openly express themselves and resolve questions about possibly taboo topics related to mental health and suicide.

Project Clan includes both informational and interactive features, ranging from traditional suicide prevention strategies (e.g., a chat with a psychologist, emergency phone hotline, and tips) that seek to reduce barriers to access quality, useful, and evidence-based information and rapid professional assistance, to components designed to increase interactions between participants and promote a sense of belonging and connection with the other Clan members (see Table 1).

**Table 1:** Description of the Intervention.

**Component**	**Description**
**INFORMATIONAL STRATEGIES**	
Tips	Evidence-based, professionally approved information, adapted by adolescents, on how to find help for oneself (from self help to crisis management) and how to help others, on risk and protective factors, and other life topics (study habits, alimentation, public speaking, recycling, exercise, safe sexual practices, and general health, etc.).
Myths vs. Realities	A section to demystify common misconceptions and stereotypes about adolescent mental health and suicide and other relevant topics.
Fact sheets	Infographics and didactic information to share useful dates about mental health conditions, bullying, suicide, etc.
Narratives	A space for videos and experiences of peers.
News and calendar	Relevant local, regional, and international news stories and cultural events will be shared.
**INTERACTIVE STRATEGIES**	
Avatars	To maintain anonymity, members will be able to create their profile and have option to a wide range of gender neutral personalization options for their avatars.
Challenges and Points	To encourage participation and promote personal and community development, members of Clan will be offered a series of challenges (categorized into personal, social, and cultural), and as they complete these challenges, and participate in the other activities of the platform, they will receive “points,” which will also them access to more avatar features, for example, or other benefits.
Forums	There will be multiple forums on a range of topics (music, movies, sports, anime, politics, news, tv shows, etc.), and members can create new topics. A central form will be focused specifically on mental health and wellbeing, and members will be able to ask the counselor specific questions.
Individual chat	Members who wish to reach out to counselor on an individual basis may do so through a private chat option.
Emergency hot line	There will be a telephone number available to call in cases of emergency (members feel they are a danger to themselves or others). This number will connect to an already operational suicide prevention hotline.
Free expression wall	There will be a central space on the platform in which members can anonymously and freely express themselves (akin to the graffitied wall of a bathroom stall).
Mood monitor/Self-evaluation	Each time they log in, members will have the option to respond a quick survey about their mood (with a range of faces and space to respond with words, if they wish).
Feedback	Members will also have the ability to send their feedback of the platform and offer suggestions for its improvement.

To develop the design concepts for the web-based platform and mobile application—including the logo, color schemes, figures, avatars, typeface, and general style—a visual artist and graph designer (FS) was incorporated into the research team. He worked closely with the “group of experts” to create a visual concept for Project Clan that was esthetically attractive, resonated with the adolescent population, and which reflected the principles of the intervention.

During the 3-month intervention, the platform will be continuously monitored by two trained psychologists, who will be serve as “counselors” and be available to answer community questions and provide support on an individual basis, as well as ensure that the rules of the community are followed (such as respect, tolerance, and confidentiality); the specific rules will be established by the participants themselves, at the beginning of the intervention. Finally, adolescents from the “group of experts,” who played a central role in the creation of the intervention model, as noted above, will have access to the platform to facilitate discussions especially in the initial stages of the intervention.

### Intervention arm

Each participant randomized to the intervention group will be assigned a user name and password to access both the static and interactive components of Project Clan, and they will have complete anonymity, until the counselor supervising the platform identifies behaviors associated with suicide risk and proceeds to follow an established emergency protocol that is further explained below (see Ethical Considerations).

### Control arm

Participants in the control group will also be assigned a username and password to access the website, but they will be met with a user interface that only displays a space to answer the corresponding assessments. In addition to the introductory presentation, they will be given a brochure with information regarding adolescent suicide and wellbeing and tips with regard to seeking help and assisting others. This will include the contact information for a telephone hotline, to ensure they can receive professional help if needed.

### Fidelity assessment

Fidelity is defined as the degree to which an intervention or program is delivered as planned (29). Following the recommendations of the NIH Behavior Change Consortium (30), fidelity will be evaluated throughout the trial through a number of approaches. First, a manual containing the principles and main strategies of the intervention will be developed. Second, on the basis of this manual, a brief scale will be designed and utilized to assess the main components of the program at various moments of the intervention (before and during implementation). Third, qualitative methods, including in-depth interviews and focus groups with deliverers, peers, and participants, will be conducted in order to complement the information collected by the fidelity scale. Finally, a thematic matrix will be developed with the goal of integrating both quantitative and qualitative findings.

### Outcomes and measures

The study's primary outcome is suicidal ideation measured with the Okasha Suicidality Questionnaire at the 2-month follow-up (see Table 2 below). Secondary outcomes include other negative psychological outcomes (e.g., stigma, depression, anxiety) as well as a number of protective psychological and social factors. All measures have been validated for use in Chile.

**Table 2:** Outcome and Measures.

**ASSESSMENT *Domain/Test***	**Time**	**Description of instrument**
**SOCIODEMOGRAPHIC**		
	5 min	Age; gender; education level; family income; school attended (public vs. private).
**PRIMARY OUTCOMES**		
Suicidality/ Okasha Suicidality Questionnaire (31)	2 min	Self-administered instrument exploring ideation and beliefs about suicide. Previously linked to suicide intent, depression, despair, low-self-esteem, impulsivity, and low social support. Item is sensitive to identifying immediate risk for suicide attempt. 4 items (scale 0–3; scale range = 0–12). Excellent internal consistency (Alpha = 0.89) & sensitivity.
**SECONDARY OUTCOMES**		
Self-Esteem/Coopersmith Self- Esteem Inventory (32)	10 min	Self-report scale on self-esteem among youth and adolescents in personal and social context. 58 items (scale 0–1). Good internal consistency (Alpha = 0.86) & construct validity.
Impulsivity/ BIS-11 (33)	7 min	Self-report scale assessing cognitive, motor, and not planned impulsivity. 30 items (scale 0–4). Excellent internal consistency (Alpha = 0.87) & specificity.
Self-Efficacy/ General Self-Efficacy Scale (34)	4 min	Self-report scale assessing self-efficacy among youth cross a number of daily stressors. 10 items (scale 1–3). Good reliability (Alpha = 0.79).
Coping Strategies/Coping Across Situations Questionnaire (35)	5 min	Self-report scale assessing stress coping strategies among youth. 16 items (scale 1 –5). Good reliability (Alpha = 0.63).
Social support/Perceived Social Support Scale (36)	5 min	Self-report scale assessing emotional help and advice among youth. 12 items (scale 1–5). Good reliability (Alpha = 0.86).
Social Skills/EHS (37)	8 min	Self-report scale assessing social skills through self-expression of anger or compliance in difference scenarios. 33 items (scale 1–4). Excellent reliability (Alpha = 0.91) & construct validity.
Depression, Anxiety, & Stress/DASS-21 (38)	6 min	Self-report scale assessing depression, anxiety, and stress symptoms. 21 items (scale 0–3). Good reliability (Alpha = 0.87) & construct validity.
Stigma/Discrimination and Devaluation Scale (39)	6 min	Self-report scale assessing awareness of stereotyping attitudes toward mental illness (12 items) (scale 0–3). Good reliability (Alpha = 0.82) & construct validity.

A third set of outcomes will evaluate implementation outcomes such as adoption, utility, and functionality of the web-based suicide intervention. “Adoption” indicators will be assessed via website metrics, for example, the number of times users sign in and the amount of time spent on the platform or with the application each day; the frequency and time spent on interactive platform connections; and the use of mental health web resources. “Utility” and “Functionality” will be measured via participants' perspectives immediately post-intervention, which will be gathered through a survey administered through the study platform. Survey questions will assess the following: a) ease of web access; b) satisfaction with the presented contents regarding wellbeing, suicide prevention, and the promotion of protective factors; c) ease of use of the interactive platform; and d) possible improvements for future applications. These assessments will facilitate potential modifications to be incorporated in the platform in future endeavors.

### Sample size and power calculation

The minimum sample size (*n* = 428) was derived based on a study conducted by van Spijker et al. (11), who also evaluated a web-based suicide intervention for adolescents with depression and suicidal ideation. van Spijker's study estimated an effect size of d = 0.35 according to Cohen's parameters (an effect size that is characterized as “small to moderate”) to identify differences between groups on suicidal ideation. In order to detect an effect size of 0.35 with α = 0.05 and b = 0.95, 214 subjects per arm are needed. Even if 20% loss to follow-up occurs at the 2-month follow—up assessment in each group (which is much larger than what was observed in previous studies) (12), a sample size of 173 subjects per group with α = 0.05 and b = 0.90 will be still able to detect an effect size of 0.35 between groups. Considering the large group of potential participants for this study (see Recruitment and Screening), reaching the proposed sample size should be feasible.

### Data analysis plan

An “intention to treat” approach will be employed to conduct main analyses. The assumptions of parametric statistical analysis will be tested (normal distribution, and homogeneity of the error variances), and descriptive and bivariate analyses on primary (suicidal ideation) and secondary (e.g., depression) outcomes will be performed. Our main analysis considers to test for differences on the primary outcome (suicidal ideation) between the intervention and control groups at the 2-month follow-up by conducting lineal and multiple regression models. A similar procedure will be conducted to test for differences on secondary outcomes with and without controlling for a set of covariates that include age, gender, education, and family income. Since 3 time-point assessments will be conducted (baseline, post-intervention, and 2-month follow-up), multilevel modeling (MLM) for repeated measures will be used to estimate time trends by comparing the effect of the intervention on primary and secondary outcomes by evaluation time. Further analyses will include “per protocol analysis” based, for instance, on how many components of the intervention participants utilized, as well as the frequency of use of the platform (how many time a day or a week) and patterns of use.

### Ethical considerations

Pertinent study documents (e.g., research protocol, instruments, informed consent, and informed assent forms) were approved by the Ethics Committee for Human Subjects Research of the Faculty of Medicine, of Universidad de Chile. In the informed consent and assent process, potential participants, and their parents or guardians, if they are under the age of 18, will be informed about the possibility to participate in this study and its objective and procedures. All participants will be informed of the voluntary nature of this study and the possibility to end their participation without affecting in any way their standing or access to education at their school, or access to mental health care, should they require it. Additionally, confidentiality will be guaranteed, to ensure data integrity and the protection of personal information. All in-person evaluations will be conducted by mental health professionals, who will receive training on human subjects protection. Participants will be given a copy of the consent form, which will contain the contact information for the Principal Investigator, in the event of any concern. Information provided by the participants will be stored in the Data Coordinating Center at Faculty of Medicine, Universidad de Chile. This data will be only handled by research staff, who will be blinded to the status (intervention or control) of each participant.

None of the activities of the project will have a direct economic cost to the participants. A system for reporting and evaluating adverse events will be established (including suicidal ideation and suicide attempts). This will consider the notification to the Ethics Committee, school authorities, and other relevant institutions, such as mental health services, if a suicide attempt occurs. This notification will be made immediately (within 24 h) and the process will be directed by the principal investigator (RA). All research team members (creators of the intervention, interviewers, counselors, “expert group” teenagers) who interact with the participants of this study will be trained in the detection and reporting of ideation/suicide attempts. Permanent contact with the Chilean Ministry of Health will be maintained, and they will provide support in handling crisis situations and linking participants to emergency mental health services emergency if necessary.

Finally, this study complies with the NIH standards and timeline for dissemination of results. Accordingly, the study has been registered at ClinicalTrials.gov by the principal investigator (RA). He will be the responsible for the accuracy of the record and for updating it at least every 12 months, as required. Also, aggregate results will be reported no later than 1 year after the clinical trial completion date.

## Discussion

Adolescent suicide has recently gained increased public attention, given the growing suicide rates reported worldwide. However, the response from health services and educational system is still insufficient. The lack of programs tailored to target this population is even worse in LMICs, where significant gaps in mental health services for youth have been documented (3). A way to address such gaps is through the use of ICTs, in developing and implementing programs that enhance protective factors against suicidal behavior. Some evidence from HICs has already shown the potential benefits of such programs but further research is needed, especially in settings with restrained resources. Providing local evidence is crucial for persuading policy makers and other stakeholders, which is critical to ensure that these programs, in case they prove to be effective, could be widely disseminated and scaled up.

Accordingly, we have described a novel ICT-based program aimed to target youth suicide in Chile. This is the first RCT of such a program in Latin America, and to our knowledge, the first of its kind in any LMIC. Given the increasing use of technological devices in LMICs, this program has the potential to inform future initiatives that seek to introduce ICT—based interventions in those countries.

## Author contributions

FM, ET, SS, and RA designed and wrote the study protocol. FM, ET, and SS conducted the literature searchers and wrote the first draft of the manuscript. MB, LY and RA provided valuable feedback in terms of methods, description of the intervention and ethical considerations. All the authors made substantial contributions, and reviewed and approved the final manuscript.

### Conflict of interest statement

The authors declare that the research was conducted in the absence of any commercial or financial relationships that could be construed as a potential conflict of interest.
